# Secondary purulent infections of the elbow joint: a retrospective, single-center study

**DOI:** 10.1186/s12891-020-3046-6

**Published:** 2020-01-18

**Authors:** Valentin Rausch, Alexander von Glinski, Thomas Rosteius, Matthias Königshausen, Thomas A. Schildhauer, Dominik Seybold, Jan Gessmann

**Affiliations:** Department of General and Trauma Surgery, BG University Hospital Bergmannsheil, Bürkle-de-la-Camp-Platz 1, 44789 Bochum, Germany

**Keywords:** Septic arthritis, Elbow empyema, Osteomyelitis, Elbow, Infection

## Abstract

**Background:**

Septic arthritis of the elbow joint is a rare condition. Limited data is available on infections of the elbow joint following trauma or prior surgery on this joint. The aim of this study was to describe the etiology, comorbidities, bacterial spectrum and therapy of secondary purulent elbow infections.

**Methods:**

Patients treated in our hospital were selected through retrospective chart review between 2006 and 2015. We included all patients with an empyema of the elbow after a trauma or surgical intervention on this joint. 30 patients between 26 and 82 years (mean: 52.47) were included.

**Results:**

Seven patients (23.3%) were female, 23 (76.7%) male. 22 patients (73.3%) had a history of trauma, eight (26.7%) had prior elective surgeries on their elbow. Between one and 25 surgeries (mean: 5.77) were necessary for treatment. In nine patients, debridement and synovectomy were sufficient, eight patients (26.7%) received resection of the elbow joint. One patient was treated with a chronic fistula. In 18 patients (60%), cultures of aspiration/intraoperative swabs were positive for *Staphylococcus aureus*, four of these were methicillin-resistant. Four patients (13.3%) had positive cultures for *Staphylococcus epidermidis*, in five patients (16.7%) no bacteria could be cultured.

**Conclusions:**

Secondary infections of the elbow joint are a rare disease with potentially severe courses, requiring aggressive surgical treatment and possibly severely impacting elbow function. *Staphylococcus aureus* was the most common bacteria in secondary infections and should be addressed by empiric antibiotic treatment when no suspicion for other participating organisms is present.

## Background

In general, septic monoarticular arthritis has an annual incidence of 2–5/100,000 in the USA with higher rates in patients with rheumatoid diseases or prostheses of the respective joint [1]. Missed treatment can quickly lead to irreparable defects of the infected joint. Despite early therapy in the course of the disease, some degree of functional impairment remains in the majority of the reported cases [2, 3]. Thus, infected joints are considered to be an orthopedic emergency, with correct diagnosis and rapid treatment being of utmost importance. However, diagnosis of an infected joint can be challenging, since neither laboratory investigations nor imaging of a joint provides sufficient specificity or sensitivity for proof of a joint infection [4]. The most important risk factors for septic arthritis are thought to be higher age as well as underlying diseases such as rheumatoid arthritis, diabetes mellitus, recent trauma or osteoarthritis [1, 5]. The highest mortality can be observed in poly-articular infections, usually occurring in patients with systemic diseases such as rheumatoid arthritis [6, 7]. Although irrigation and antibiotic treatment has been shown to be sufficient in most patients, more radical treatment such as local antibiotic treatment with use of antibiotic beads or even resection of the joint might be inevitable in some cases [4].

In particular, septic arthritis of the elbow joint remains a rare but devastating condition. The elbow is the third most affected joint in mono-articular septic arthritis, in a UK-based study it was shown to be present in about 9% of the cases [5]. However, specific epidemiological data and treatment options in septic arthritis of the elbow joint are limited [8, 9]. As in other joints, general treatment recommendations include irrigation (open or arthroscopic), drainage of the joint and antibiotic therapy [8].

The aim of this study was to report our clinical experience with secondary infections of the elbow joint and to derive treatment recommendations. Bacterial spectrum, necessary treatment and outcome are reported. We hypothesized that a significant proportion of the patients could not be sufficiently treated with irrigation of the joint alone and more radical procedures would frequently be required to treat this disease.

## Methods

We retrospectively reviewed the medial charts of our institution between 2005 and 2016 for patients with infectious diseases around the elbow joint.

In this study we included patients with a grossly purulent joint that could be seen in arthroscopy, arthrotomy or after puncture of the joint, regardless of the type of operative procedure performed for treatment of the infection. All patients with a trauma to the elbow or prior elective operative procedures to the elbow joint except total elbow arthroplasty or radial head arthroplasty were included. Patients with sole radiologic or microbial findings without active purulence were not included in this study to avoid false-positives. Patients suffering from immunodeficiency (acquired or due to an underlying disease such as rheumatoid arthritis or HIV) were excluded. Using these criteria, we found 32 patients suffering a septic arthritis of the elbow after a trauma (*n* = 24) or after elective surgery of the elbow joint (*n* = 8) in the relevant period.

We then reviewed the medical charts of the patients for the following features: age, sex, comorbidities, mortality, laboratory parameters (C-reactive protein (CRP), white blood cell count (WBC)), findings in microbial cultures, presentation after onset of the symptoms, number of surgical procedures, definitive therapy and documented range of motion before dismissal.

Number of surgical procedures, age, outcome and laboratory parameters were compared between seropositive (with positive bacterial culture) and seronegative (without positive bacterial culture) patients. To test for significant differences, the Mann-Whitney-U test was used.

**Fig. 1:**
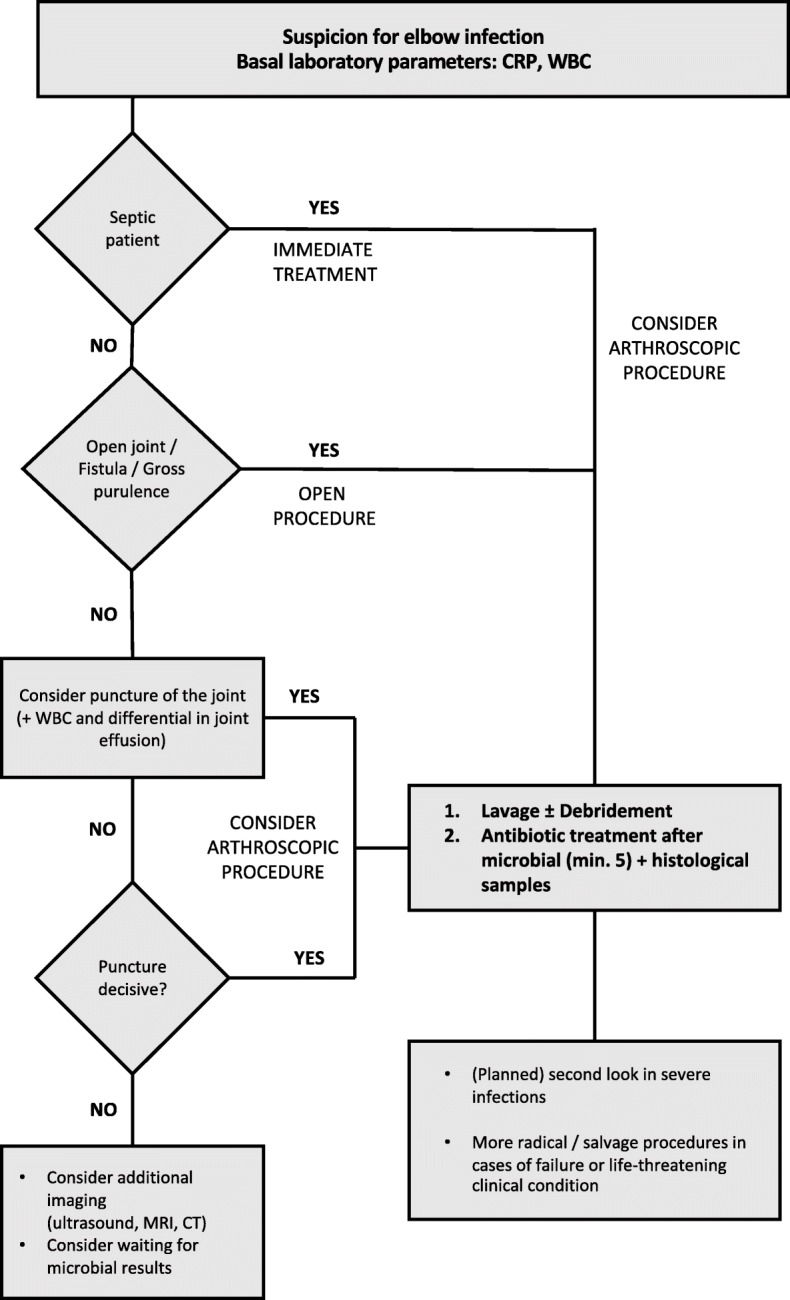
Flowchart for the treatment of (non-periprosthetic) secondary infections of the elbow

Patients where a (non-periprosthetic) septic arthritis is suspected are generally treated based on the following proposed staged protocol of our institution (Fig. 1). This protocol has been derived from and is based on published research and treatment recommendations [10]. When presenting with clinical symptoms (such as pain, swelling, redness, warmth of the affected area), patients are investigated for elevated laboratory serum-parameters (CRP and WBC). If the patient is in a critical condition, the patient receives immediate operative treatment. If possible, the lavage of the joint is performed arthroscopically. If the patient presents with an open joint, gross purulence or a fistula, lavage and debridement are performed as an open procedure. If laboratory parameters are not indicative and clear clinical signs are missing, a puncture of the joint with measurement of the WBC and a differential in the joint aspirate can help determine whether the joint is infected. In cases with a remaining clinical suspicion, further imaging studies (i.e. ultrasound, MRI, CT) can be used to find further signs for a joint infection and is routinely used in such cases. If the clinical condition allows delay of treatment, we consider waiting for microbial results on a case by case basis. Also, an (arthroscopic) lavage and debridement offers the possibility of more sensitive microbial and histological samples.

In case of revisions of recent operative procedures, the chosen operative approach to the elbow is usually the same as to the primary procedure. If an open procedure is otherwise favored, we use the Kocher-approach for optimal exposure of lateral pathologies. On the medial elbow, we usually use the Hotchkiss-approach, on the dorsal elbow a paramedian incision is favored. Wound closure is performed with monofilament sutures and drains are administered on the surgeon’s discretion. In patients without external fixation, immobilization is generally performed using a long arm cast. We generally administer calculated intravenous antibiotic treatment for an infection with *Staphylococcus aureus* (for example Cefazolin 2 g every 8 h) if indications for other microbial organisms are missing. In general, if patients show signs of an immunodeficiency, we consider a broader antibiotic treatment aiming against *methicillin-resistant Staphylococcus aureus (MRSA)* or gram-negative bacteria. Second look operations are planned in critical infections approximately two days after the initial surgery. Intravenous antibiotic treatment is adapted to the results of the microbial analysis once available. All microbial cultures are held for 14 days including stains for fungus and acid fast bacteria before being confirmed negative.

In persistent infections, additional surgeries are performed depending on the intraoperative findings. More radical procedures such as resection of joint surfaces are indicated if the elbow joint is destroyed or a life-threatening disastrous infection make the immediate complete removal of the infected joint necessary.

Application of antibiotics is changed from intravenous to oral administration after normalization of laboratory parameters. We recommend antibiotic treatment for at least 6 weeks after the initial surgery.

## Results

Of a total of 30 patients, seven (23.3%) were female, 23 (76.7%) were male (Table 1). Mean age at admission was 52.47 years (range: 26–82). All patients were treated as inpatients in our hospital. In 22 patients (73.3%), a history of trauma was reported, eight patients (26.7%) received prior elective surgery on their elbows. Of the patients with a history of trauma, four had an infected non-union. Ten patients suffered an early infection after a trauma or fracture of their elbow. In these cases, an early removal of the osteosynthesis was performed in five cases. Eight patients suffered an infection > 4 weeks after their initial surgery. In five patients, besides debridement and lavage of the joint, osteosynthetic material was removed. Additionally, four patients (13.3%) had diabetes mellitus, nine patients (30%) suffered from high blood pressure (Table 2).

**Table 1:** clinical data of patients

Sex	8 female (25%)
Age	49.75 (9–82)
Post trauma	8 (25%)
Post surgery	24 (75%)
CRP^a^	7.03 mg/dl (0.1–43.1)
WBC^b^	10.79 /nl (5.3–20.7)

^a^*CRP* C-reactive protein

^b^*WBC* White blood cell count

**Table 2:** pre-existing conditions in patients with septic arthritis of the elbow

Disease	Count
High blood pressure	9
Diabetes mellitus	4
Hypothyroidism	2
Hepatic insufficiency	2
Spinal chord injury	2
Renal insufficiency	1
Hyperuricemia	1
Atopic dermatitis	1
Periarticular ossifications	1

We observed the following treatment methods: Nine patients (30%) could sufficiently be treated with initial debridement and synovectomy of the joint. A resection arthroplasty was necessary in eight patients (26.7%), a reconstruction of a soft tissue defect with a myocutaneous, local flap or a skin graft was necessary in four patients (13.3%). In one case, a disastrous infection resulted in amputation at the distal humerus. Local antibiotics with use of a gentamicin-collagen sponge were administered in 5 patients (16.7%). Following treatment, 29 patients (96.7%) left the hospital after being cured of the infection. The remaining patient (3.3%) received a chronic fistula and was dismissed with a chronic infection of the elbow. In total, 10 patients (33.3%) received a salvage procedure (chronic fistula, amputation or resection arthroplasty) to treat the infection. A median of five procedures per patient (range: 1–25; SD ±4.9) was performed. No patient died of their disease during their hospital stay. Patients were followed for a mean follow-up of 30.4 month (range: 8 day – 12.8 years; SD: ±34.8 month) after first admission to the hospital for treatment of the infection. Seven patients were lost to follow-up (23.3%). In 24 patients the arc of motion was measured at dismissal of the hospital or at the latest follow-up: They reached a mean arc of motion of 76.3 °.

**Fig. 2:**
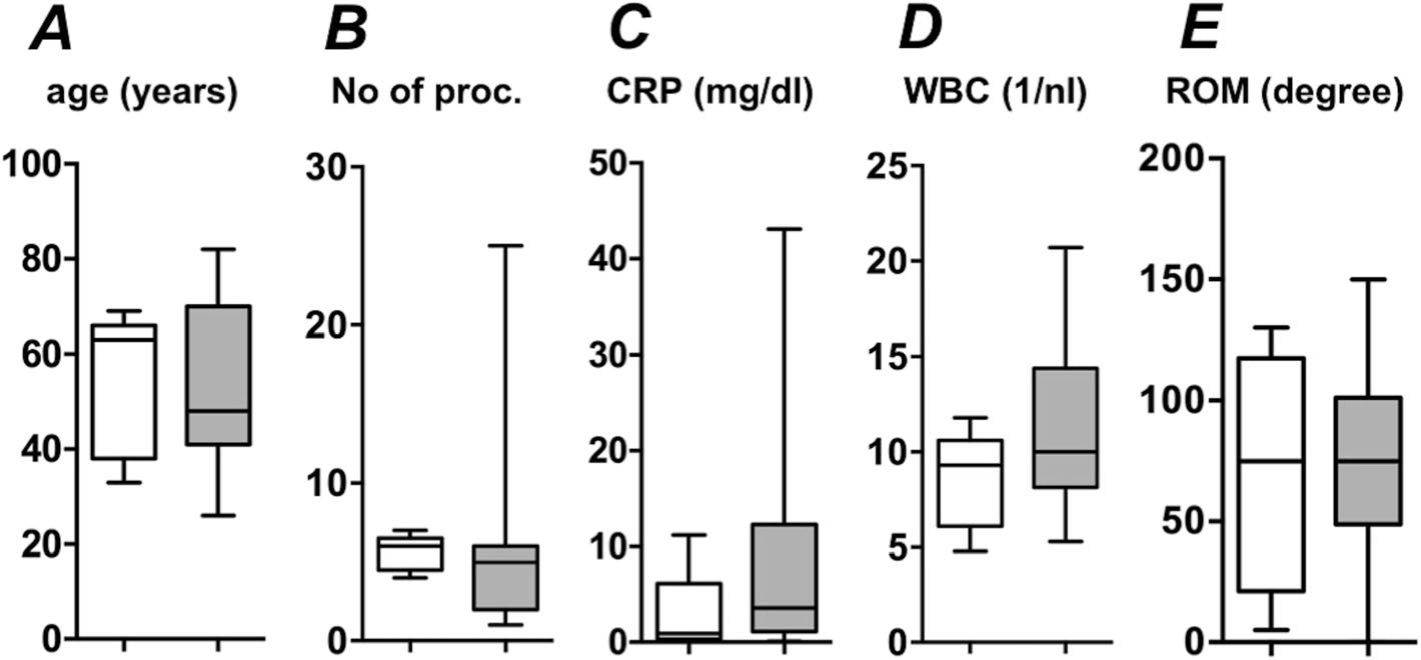
Laboratory and clinical parameters in seronegative and seropositive elbow infections. **a** age, **b** number of procedures, **c** C-reactive protein (CRP), **d** white blood cell count (WBC) and **e** range of motion (ROM) in seronegative (white background) and seropositive (grey background) elbow infections

A positive microbial culture of the puncture or a positive intraoperative swap could be found in 25 patients (83.3%): Most cases were positive for *Staphylococcus aureus* (18 patients, 60%) from which four (13.3%) were methicillin-resistant. *Staphylococcus epidermidis* could be detected in another four patients (13.3%). When comparing patients with a seropositive (positive bacterial culture) and seronegative (negative bacterial culture), no significant differences could be found in regard to age (*p* = 0.73), number of operative procedures needed (*p* = 0.49), CRP (*p* = 0.62) or white blood cell count (*p* = 0.31) (Fig. 2).

## Discussion

In this study, we reviewed patients with a purulent secondary elbow infection in particular for their mortality, definitive therapy, outcome and findings in bacterial cultures.

While we observed no mortality related to secondary elbow joint infections, we describe severe courses of this disease requiring multiple interventions in several cases, in one case even an amputation of the forearm. Three patients needed > 10 procedures, since the infection recurred after the initial treatment, in one patient this led to a resection arthroplasty and chronic fistula with 25 procedures in total. We found a high rate of 28% of patients receiving radical salvage procedures such as resection arthroplasty of the joint as treatment of the infection, underlining the severity of a septic arthritis of the elbow joint. In line with these findings, we observed a high median impairment regarding the range of motion of the respective elbow. The leading organism in our study causing the elbow joint infection was *Staphylococcus aureus*.

In general, septic arthritis of non-rheumatic patients without a prosthesis is a rare disease. Weston et al. found infections of the elbow and shoulder joint in poly-articular septic arthritis to be associated with a higher mortality in their multivariate analysis [5]. In contrast, mortality of a monoarticular infection of the elbow joint in this study was determined 5%, while the overall mortality rate was 11.5% in septic arthritis [5]. At first glance, our data is not in line with these findings: In our collective, no patient died from a secondary infection to the elbow joint during the period under review. However, patients suffering from diseases that affect the immune system (due to their medication or the underlying condition) were excluded in our collective. We believe the exclusion of patients treated with immunosuppressive therapy or underlying systematic diseases (such as patients treated for rheumatoid arthritis) accounts for the differences in our results as survival rates of previously healthy patients might differ substantially from those suffering from immunodeficiency. Therefore, we consider our data not to be contradictive to the aforementioned study.

We could also show septic arthritis to be a possible complication of previous elective surgical procedures close to the joint as is common in the surgical treatment of a tennis elbow or chronic bursitis, for example. This is in line with a study of Moon et al., who also presented otherwise healthy patients affected by septic arthritis of the elbow joint [8].

To our knowledge, the only study reporting specifically about septic arthritis in the elbow joint was performed by Mehta et al. in 2006 [9]. In their study, the authors focused on patients with hematogenous septic arthritis and therefore specifically excluded all patients with open joints or previous surgical procedures on the elbow, making a direct comparison of our collectives difficult. Our findings in bacterial cultures were however in accordance with the findings of bacterial cultures in septic arthritis described in literature and in particular Mehta et al’s study [4, 9].

Given the severity of septic arthritis of the elbow joint shown in our study, great care must be taken not to undertreat patients with infections to the elbow joint, since misinterpretation of early symptoms (pain, swelling, warmth at the affected area) and thus missing early diagnosis can have disastrous effects. When evaluating patients with septic arthritis, diagnostic accuracy of laboratory values such measurement of CRP or WBC alone is insufficient [11, 12]. Another powerful tool in cases with a suspicion of a septic arthritis could be the WBC and percentage of polymorphonuclear cells in joint aspirate [11, 12]. This parameter can be determined even if Gram stains or bacterial cultures are not (yet) available. Margaretten et al. propose a leukocyte count of greater than 50,000 cells /mm^3^ as a diagnostic predictor for septic arthritis [11, 13]. However, a low WBC in the joint aspirate cannot rule out a septic arthritis [11].

Infections to the joint can also be present in cases of seronegativity. Gupta et al. found the outcome in patients with seropositive septic arthritis comparable to those with a high clinical suspicion but seronegative septic arthritis [14].

Therefore, patients where septic arthritis is suspected should immediately be treated operatively with drainage of the purulent effusion, surgical debridement followed by antibiotic treatment [4, 15, 16]. However, evidence is missing in regard to the application regimen of antibiotic treatment: In general, intravenous application of antibiotics is recommended [4]. Empiric antibiotic treatment should generally focus on *Staphylococcus aureus*. However, treatment strategy should be adapted according to local resistances and include treatment of *methicillin-resistant Staphylococcus aureus (MRSA)* or other (resistant) organisms if indications for resistance are observed.

In our practice, we use intravenous Cefazolin as calculative first-line treatment for septic arthritis if an indication for a broader antibiotic treatment is missing (Fig. 1).

As for the operative technique, there seems to be no clear indication of a superiority of arthrotomy over arthroscopic drainage or needle-aspiration in the literature [8]. In any case, arthroscopic drainage could be shown to be safe in elbow infections with the advantage of a minimal-invasive procedure while at the same time allowing an assessment of the joint [8]. More radical procedures as seen in our study were necessary in patients with life-threatening conditions call for an immediate and complete removal of the septic arthritis. Destruction of the elbow joint after open fractures as well as an osteomyelitis on the humerus or the radius/ulna can require a resection of the joint. Life-long antibiotic treatment as suppression therapy or creation of a fistula as a last resort for non-curable infections remain the exception but may unfortunately be necessary in individual cases. In our proposed staged protocol, we do not consider the time delay between the index surgery or trauma and revision surgery as indicative for a specific treatment algorithm. Operative therapy in these patients is highly individual and a staged protocol can only serve as orientation for such treatment. In general, we do not recommend radical treatments solely based on time delays of secondary joint infections but suggest basing such decision on the local extent of the infection. Therefore, if an infection is strictly limited to the elbow joint, we primarily perform an (arthroscopic) debridement and lavage of the joint.

Our study has several limitations: We performed a retrospective observational study of patients treated with secondary septic arthritis in our institution. Long-term outcome after dismissal of the patients could therefore not be described. Also, due to the structure of the German health care system, patients are usually referred to a maximum care hospital, from a primary care facility or by the emergency services when patients are in a poor general condition. Milder cases might be treated in primary care facilities such as general or regional hospitals or in outpatient medical-care centers with a less severe outcome and fewer comorbidities. Therefore, our results could be somewhat distorted in regard to the severity and the outcome. Our results might hence not be comparable to a potential larger collective also including milder cases of elbow infections. However, due to the infrequency of the disease, a prospective single-center study to describe influencing factors in this disease might not be feasible. A larger study in a multi-center design with inclusion of primary care facilities might overcome these limitations. Furthermore, cases with sole microbial or typical radiological signs were disregarded in this study, leading to a selection bias to more severe cases where turbid fluid was detected.

## Conclusions

Secondary purulent infections of the elbow joint are a rare disease with potentially severe courses requiring aggressive surgical treatment and which may lead to a severe impact on elbow function.

To avoid worse outcomes, thorough diagnosis and rapid treatment with empiric antibiotics and drainage should be performed when secondary infections of the elbow joint are suspected. Empiric antibiotic treatment should focus on *Staphylococcus aureus* when no suspicion for methicillin-resistance or other participating organisms is present but should be adapted accordingly if this assessment is changed. Although evidence is scarce, we recommend arthroscopical drainage or arthrotomy if a purulent infection is present to ensure sufficient lavage and possibly resect affected tissue.

## Data Availability

The datasets used and/or analysed during the current study are available from the corresponding author on reasonable request.

## Abbreviations

CRP: C-reactive protein
MRSA: Methicillin-resistant *Staphylococcus aureus*
ROM: Range of motion
WBC: White blood cell count
